# Sex-Related Disparities in the Resting State Functional Connectivity of the Locus Coeruelus and Salience Network in Preclinical Alzheimer’s Disease

**DOI:** 10.3390/ijms242015092

**Published:** 2023-10-11

**Authors:** Yoo Hyun Um, Sheng-Min Wang, Dong Woo Kang, Sunghwan Kim, Chang Uk Lee, Donghyeon Kim, Yeong Sim Choe, Regina E. Y. Kim, Soyoung Lee, Hyun Kook Lim

**Affiliations:** 1 Department of Psychiatry, St. Vincent’s Hospital, College of Medicine, The Catholic University of Korea, Seoul 06591, Republic of Korea; 2 Department of Psychiatry, Yeouido St. Mary’s Hospital, College of Medicine, The Catholic University of Korea, Seoul 06591, Republic of Korea; 3 Department of Psychiatry, Seoul St. Mary’s Hospital, College of Medicine, The Catholic University of Korea, Seoul 06591, Republic of Korea; 4 Research Institute, Neurophet Inc., Seoul 08380, Republic of Korea; 5 Department of Psychiatry, Brigham and Women’s Hospital, Boston, MA 02115, USA; 6 Department of Psychiatry, Harvard Medical School, Boston, MA 02115, USA

**Keywords:** salience network, locus ceoruleus, Alzheimer’s disease, preclinical, sex

## Abstract

Recent studies have demonstrated the pivotal role of locus coeruleus (LC) and salience network (SN) resting state functional connectivity (rsFC) changes in the early stage of Alzheimer’s disease (AD). Moreover, sex has been a crucial point of discussion in understanding AD pathology. We aimed to demonstrate the sex-related disparities in the functional connectivity (FC) of the SN and LC in preclinical AD. A total of 89 cognitively normal patients with evidence of amyloid beta (Aβ) accumulation ([18F] flutemetamol +) were recruited in the study. A seed-to-voxel analysis was conducted to measure the LC and SN rsFC differences between sexes. In addition, sex by Aβ interactive effects on FC values were analyzed with a general linear model. There were statistically significant sex by regional standardized uptake value ratio (SUVR) interactions in the LC FC with the parietal, frontal, and occipital cortices. Moreover, there was a significant sex by global SUVR interaction in the SN FC with the temporal cortex. The findings suggest that there are differential patterns of LC FC and SN FC in males and females with preclinical AD, which interact with regional Aβ deposition.

## 1. Introduction

Resting state functional connectivity (rsFC) in Alzheimer’s disease (AD) has been extensively studied regarding its diagnostic power and clinical implications. One systematic review demonstrated evidence that the FC of default mode network (DMN) nodes confers a relatively high diagnostic power in identifying AD [1]. However, despite their importance in understanding AD pathophysiology, longitudinal FC disparities in the AD spectrum with regard to covariates such as education, apolipoprotein E carrier state, and sex remain elusive. Sexual dimorphism has been a rigorously studied topic in AD research, since its purported role in AD trajectory can be integral to delivering individualized prevention and treatment to AD patients [2]. Differential activational and organizational effects of sex hormones between the male and female group played critical roles in paving distinctly disparate pathways in AD pathogenesis [3,4]. Moreover, recent multi-omics studies have pointed to the sexually different molecular pathways in neuroinflammation and bioenergetics metabolism [5].

The National Institute of Aging/Alzheimer’s Association defines preclinical AD as normal cognition with evident amyloidosis of the brain [6]. RsFC studies in preclinical AD have important clinical implications, since they predate symptom onset [7]. Previous rsFC studies delineated that, like the AD and mild cognitive impairment (MCI) populations, preclinical AD patients show a decreased precuneus and hippocampal FC [8]. Moreover, the DMN, salience network (SN), and frontoparietal control network synergistically interact with Aβ deposition in predicting cognitive decline in preclinical AD. Phases of hyperconnectivity and hypoconnectivity have been noted in the DMN and SN according to neocortical tau levels [9]. Despite the clinical implications of rsFC studies in the preclinical AD population, studies including sex as a biological variable are scarce. One study demonstrated that men with normal cognition (NC) exhibited a lower DMN FC, but sex by amyloid deposition interactions were not significant [10]. Moreover, female APOE4 carriers with NC displayed a reduced hippocampal FC [11]. However, those studies failed to exclusively analyze NC participants with brain amyloidosis based on the specific definition of preclinical AD. Additionally, most studies have focused on conventional networks, mostly the DMN.

The locus coeruleus (LC) has been gaining the spotlight in AD research recent years [12]. One recent study demonstrated that early LC tau accumulation was closely associated with AD progression, and that LC integrity was an incipient biomarker of subtle cognitive deterioration in preclinical AD [13]. Moreover, lower novelty-related LC activity was closely related to Aβ-related cognitive decline in cognitively normal (CN) participants [14]. Therefore, the structural, functional correlates of the LC in preclinical AD research are crucial, but limited studies are extant. Additionally, the locus coeruleus-norepinephrine system (LC-NE) system is affected by estrogen, and an intricate interplay between the LC-NE system influences blood–brain barrier integrity and neuroinflammation [15]. A higher LC FC in males has been reported [16], and a reduced LC signal intensity in females has been demonstrated in CN participants [17]. Despite the aforementioned sexual disparities in the properties of the LC, few studies have demonstrated the interactive effects of sex and Aβ deposition in AD trajectory [15,18].

Meanwhile, the SN is well-known for its function in maintaining network homeostasis through optimally responding to relevant stimuli. A recent study which adopted a machine learning method reported that abnormal connections in the SN were effective in identifying subjective cognitive decline in addition to the DMN [19]. In a 4-year longitudinal study on CN participants, FC alterations were apparent in the SN and frontoparietal network, but not in the DMN [20]. Another study reported that SN segregation and coupled glucose metabolism were evident in the CN group, while this coupled relationship disappeared as AD progressed [21]. In a recent study applying a deep learning model to resting-state functional brain network data in sex classification, resting-state data were superior to task-based data in sex classification, and the SN was the most significant in defining sex differences [22]. However, studies exploring the alterations in the SN in AD trajectory with considerations of sex are limited.

In this regard, we aimed to demonstrate the sex-related disparities in the FC of the SN and LC in preclinical AD. To the best of our knowledge, this is the first study to explain the FC of the SN and LC in preclinical AD within the context of including sex as an integral biological variable. We hypothesized that there will be significant differences in the FC of the SN and LC according to sex. Moreover, we postulated that the FC of the SN and LC will be disrupted by an interaction between amyloid deposition and sex.

## 2. Results

No statistical difference was observed in terms of age, APOE4 carrier status, education, and CERAD-K battery subscore between males and females (Table 1). All the participants were identified to be amyloid-positive, with a global SUVRPONS (SUVR of [18F]-flutemetamol, with pons as a reference region) of 0.7 ± 0.1 for both males and females. There were no significant differences in the global SUVRPONS or regional SUVRPONS between the male and female groups. The seed-to-voxel analysis demonstrated that there was no difference in the LC FC and SN FC between males and females.

**Figure 1:**
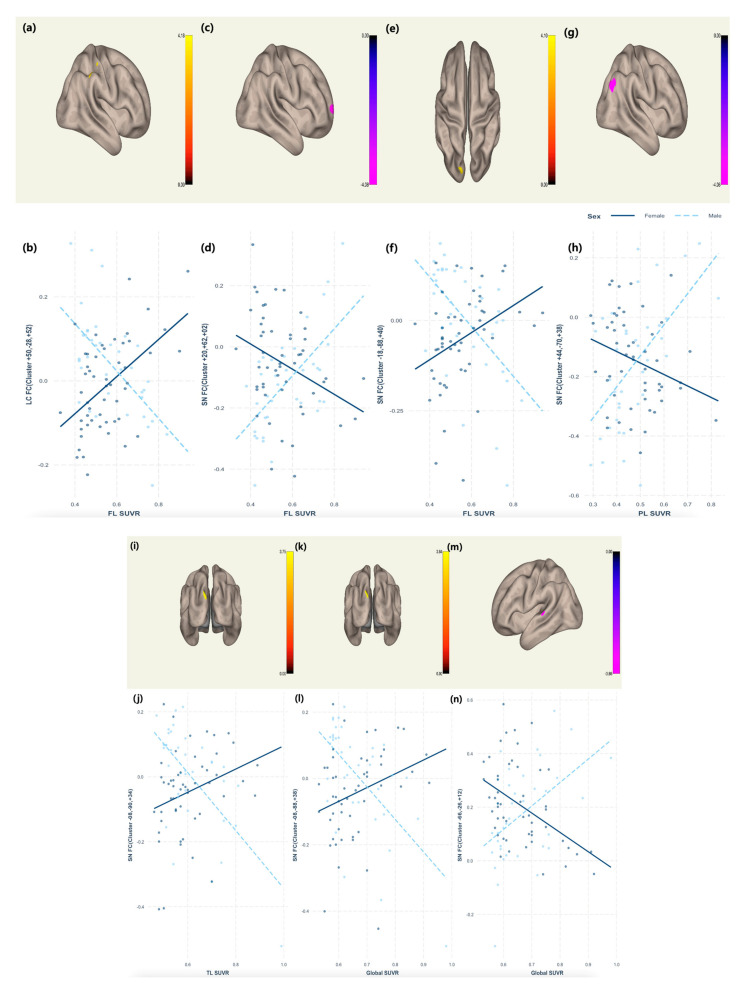
Association of the LC FC and SN FC with the interaction of sex, regional, and global SUVR, (**a**) Regions showing interactive effect of sex and FL SUVR on LC FC, (**b**) Interaction plot showing interactive effect of sex and FL SUVR on LC FC, (**c**,**e**) Regions showing interactive effect of sex and FL SUVR on SN FC,(**d**,**f**) Interaction plot showing interactive effect of sex and FL SUVR on SN FC, (**g**) Regions showing interactive effect of sex and PL SUVR on SN FC, (**h**) Interaction plot showing interactive effect of sex and PL SUVR on SN FC, (**i**) Regions showing interactive effect of sex and TL SUVR on SN FC, (**j**) Interaction plot showing interactive effect of sex and TL SUVR on SN FC, (**k**,**m**) Regions showing interactive effect of sex and global SUVR on SN FC, (**l**,**n**) Interaction plot showing interactive effect of sex and global SUVR on SN FC; LC, Locus coeruleus FL, frontal lobe; SUVR, standardized uptake value ratio; SN, salience network; PL, parietal lobe; and TL, temporal lobe.

There was a significant sex by frontal lobe (FL) SUVR interaction in the LC FC with the right postcentral gyrus and right anterior and posterior division of the SMG (standardized β coefficient = 1.0056, F = 15.08, adjusted R^2^ = 0.32, *p* < 0.01) (Table 2, Figure 1a,b). Moreover, there was a significant sex by FL SUVR interaction in the SN FC with the right frontal pole (standardized β coefficient = −1.1806, F = 9.84, adjusted R^2^ = 0.26, *p* < 0.01) (Table 2, Figure 1c,d), left lateral occipital cortex, superior division, and left occipital pole (standardized β coefficient = 1.0631, F = 9.91, adjusted R^2^ = 0.23, *p* < 0.01) (Table 2, Figure 1e,f). Additionally, there was a significant sex by parietal lobe (PL) SUVR interaction in the SN FC with the right lateral occipital cortex and superior division (standardized β coefficient = −1.4316, F = 8.40, adjusted R^2^ = 0.20, *p* < 0.01) (Table 2, Figure 1g,h). There was a significant sex by temporal lobe (TL) SUVR interaction in the SN FC with the left lateral occipital cortex, superior division, left occipital pole, and left cuneal cortex (standardized β coefficient = 1.2484, F = 10.32, adjusted R^2^ = 0.24, *p* < 0.01) (Table 2, Figure 1i,j). Lastly, there was a significant sex by global SUVR interaction in the SN FC with the left lateral occipital cortex, left occipital pole, left cuneal cortex (standardized β coefficient = 1.3968, F = 9.34, adjusted R^2^ = 0.22, *p* < 0.01) (Table 3, Figure 1k,l), left superior temporal gyrus, and left planum temporale (standardized β coefficient = −1.5993, F = 8.35, adjusted R^2^ = 0.20, *p* < 0.01) (Table 3, Figure 1m,n).

## 3. Discussion

To the best of our knowledge, this is one of the few studies demonstrating the LC and SN FC patterns in stringently classified preclinical AD with consideration for sex as a variable. According to the results, there were significant group by FL, PL, TL, and global SUVR interactions in the LC and SN FC. These findings confer important clinical implications.

There was a statistically significant sex by FL SUVR interaction in the LC FC with the right supramarginal and postcentral gyri, which are parts of the major somatosensory association cortex. Research on sex differences in the LC-NE system, especially with consideration for AD pathology, remains in its infancy. The LC-NE system is integral in honing the salience of stimuli, which may result in optimal decision making [23]. One review article proposed different thresholds for the LC of females and males in receiving stimuli that are salient and associated, which may result in the differential encoding of information between males and females—with males being centered on capturing global information and females being centered on capturing local or detailed information [24]. Increases in LC FC with the right supramarginal and postcentral gyri with an increment in the FL SUVR in females may indicate the compensatory activation of an increasing signal-to-noise ratio with the receipt of stimuli in the background of Aβ deposition in the frontal lobe. The LC-NE system is closely involved in high cognitive processes, and the LC has extensive projections to the FL [25]. FL Aβ deposition may disrupt this process and result in injuries in finely tuned cognitive processes that involve association cortices, as discussed above. Moreover, this pathological process may be disparate between females and males.

There was a significant sex by FL SUVR interaction in the SN FC with the right frontal pole, with a decreased SN FC noted as the FL SUVR increased in females. The functional impairment of the SN in AD trajectory is well-known, with altered dynamics among the SN, central executive network, and DMN [26]. Indeed, disruptions of the SN are a key feature in distinguishing AD from suspected non-AD pathology [27]. Disparities in SN components were noted between amnestic mild cognitive impairment (MCI) and Alzheimer’s disease, with amnestic MCI displaying internetwork FC components of the SN [28]. Patients with subjective cognitive decline displayed an increased insular FC with the frontal and temporal cortices in one study, and insular subnetworks were able to demarcate SCD and amnestic MCI [29]. The insular cortex was a common hub node for converting MCI, HC, and AD, indicating the critical role of this region [30].

There was a significant sex by FL SUVR interaction in the SN FC with the right frontal pole, with a decreased SN FC noted as the FL SUVR increased in females in our study. A recent study demonstrated that an insular-prefrontal FC was positively related to interoceptive accuracy in older adults [31], and sex-related differences in interoceptive accuracy were reported in a meta-analysis, with females displaying poorer scores in heartbeat-counting tasks [32]. Future studies exploring the association between the SN FC with interoceptive task measurements, along with age and sex as variables, could reveal incipient signs preceding cognitive decline in preclinical AD patients.

In addition, there were significant sex by FL, PL, TL, and global SUVR interactions in the SN FC with the left occipital cortex. A study on a mouse model with tauopathy reported that there was impaired visual plasticity, even at incipient stages of neurodegeneration [33]. A recent study on FC map alterations in subjective cognitive decline hypothesized that an increased FC of the parahippocampl gyrus to the occipital lobe was a compensatory mechanism, and a close association of the occipital cortex FC in the AD spectrum was accentuated [34]. With regard to sexual disparities, female estrogen has been known to affect the perceptual processing of visual stimuli, and a mouse study reported that the visual cortex was a region with a high sensitivity to estrogen [35]. Moreover, blood–brain barrier integrity was particularly disrupted in the occipital cortex of cognitively normal females [36]. According to our results, the SN FC with the occipital cortex was increased as the regional and amyloid Aβ deposition progressed in females, and this may represent the compensatory mechanism involved in female preclinical AD.

Lastly, there were significant sex by TL SUVR interactions in the SN FC with the superior temporal cortex. The superior temporal cortex is a part of the auditory network, and the auditory cortex and SN are important components of the somatosensory motor network. Recently, a study protocol was published with regard to investigating and unraveling the role of somatosensory integration in AD [37]. We believe our results represent the importance of somatosensory perception and integration in preclinical AD patients, in addition to our relevant proposition in the earlier paragraphs. Moreover, the crosstalk between the SN FC and the somatosensory network may be differentially disrupted between males and females.

There are several limitations that must be taken into consideration. Firstly, the study was a single-center study, which may limit the generalizability of the results. Secondly, the study was cross-sectional, which cannot represent a causal relationship between sex differences, SN FC, LC FC, and AD. Thirdly, there are the intrinsic limitations of rsFC studies which do not fully represent anatomical connectivity and can vary according to individual mental states. Thirdly, we did not include an amyloid-negative, healthy control group. Fourthly, measurements with regard to behavioral scores, lifestyle factors associated with cognitive decline [38], and correlation analyses regarding the participants’ hormone levels were not implemented. Still, our study has strengths in that the males and females were age- and education-matched, which may be construed as they had similar brain reserve and cognitive reserve capacities.

## 4. Materials and Methods

From November 2017 to August 2021, the Brain Health Center at Yeoui-do St. Mary’s Hospital, College of Medicine, The Catholic University of Korea, enrolled 89 individuals who were in the preclinical stages of Alzheimer’s disease (AD). Each of the participants underwent a Korean-adapted version of the Consortium to Establish a Registry for Alzheimer’s Disease (CERAD-K) [39], which included cognitive assessments such as a verbal fluency test, 15-item Boston Naming Test, the Korean version of the Mini-Mental State Examination (MMSE-K) [40], and evaluations of word list memory, recall, and recognition, as well as constructional praxis and recall. Cognitive normality was confirmed in all the participants, and they were all given a Clinical Dementia Rating (CDR) of 0 [41]. All the participants tested positive for amyloid beta (Aβ+) using the methods outlined in the PET scan section. None of the 89 participants had a past record of cerebrovascular incidents, signs of cerebrovascular damage, substance misuse, head injury, mental health conditions, or had been prescribed psychotropic drugs such as antidepressants, acetylcholinesterase inhibitors, antipsychotics, or sleeping aids. The research was conducted following the ethical and safety standards set by the local Institutional Review Board (IRB) of the Catholic University of Korea and the Declaration of Helsinki. Moreover, all the experimental protocols were approved by the IRB of the Catholic University of Korea. All the participants provided their written informed consent.

### 4.1. APOE Genotyping

DNA was extracted using the QIAmp Blood DNA Maxi Kit procedure by Qiagen, based in Valencia, CA, USA. Two APOE single-nucleotide polymorphisms (SNPs), rs429358 (E4) and rs7412 (E2), were determined using the TaqMan SNP genotyping tests provided by Applied Biosystems, located in Foster City, CA, USA.

### 4.2. PET-CT Image Acquisition

The production of [18F] Flutemetamol (FMM) and the collection and analysis of FMM-PET data followed previously established methods [42]. Individual participants’ MRI scans were used for aligning images and defining the region of interest (ROI) to adjust for the partial volume effects arising from the expansion of the cerebrospinal space due to brain atrophy. A standardized uptake value ratio (SUVR) at 90 min post-injection was used, with the pons ROI as a reference point. The global Aβ was calculated from the SUVR averaged from six cortical ROIs, which included the posterior cingulate cortex/precuneus, superior parietal, frontal, striatum, lateral temporal, and anterior cingulate ROIs. A SUVR value of 0.62 was established as a cut-off value to categorize the neocortical SUVR as either “high” or “low”. Any participant with an SUVR value of 0.62 or above in at least one cortical ROI was considered to be amyloid-positive [42].

### 4.3. MRI Acquisition

Imaging data were gathered at the Department of Radiology, Yeouido St. Mary’s Hospital, The Catholic University of Korea, using a 3T Siemens Skyra scanner and a 32-channel Siemens head coil (Siemens Medical Solutions, Erlangen, Germany). The T1-weighted volumetric magnetization-prepared rapid gradient echo scan sequences used the following parameters: TE = 2.6 ms, TR = 1940 ms, inversion time = 979 ms, FOV = 230 mm, matrix = 256 × 256, and voxel size = 1.0 × 1.0 × 1.0 mm^3^. A multiband gradient-echo EPI acquisition method was adopted for resting-state functional image acquisition, with TE = 86.0 ms, TR = 3100 ms, slice acceleration factor = 3, slice thickness = 2 mm, FOV = 224 mm, matrix = 128 × 128 × 29, and voxel size = 1 × 1 × 2 mm^3^. A total of 150 volumes were collected over a period of 5 min while the participant was instructed to “keep your eyes closed and think of nothing in particular”.

### 4.4. Functional Connectivity Analysis

#### 4.4.1. Preprocessing

The CONN toolbox Version 20.b was used for preprocessing and analyzing the functional imaging data, as well as computing the functional connectivity (FC) [43]. The standard CONN preprocessing sequence was adopted: functional realignment and unwarp; slice-timing correction; outlier identification; direct segmentation and normalization; and functional smoothing, but with a 4 mm Gaussian kernel, considering the small size of the LC. For the slice time correction, the retrieval of the slice timing of DICOM images for each subject was implemented, since all our functional images were obtained with the multiband gradient echo-planar imaging method. Following the end of the preprocessing, the denoising pipeline enabled the removal of confounding effects from the BOLS signal. The elimination of noise from the physiological variables and residual movement was conducted. Linear regression included all the confounders identified by ART. Individual subject data went through motion correction and linear de-trending was conducted. In addition, a default band-pass filter of [0.008 0.09] Hz was employed.

#### 4.4.2. Seed-to-Voxel Analysis

Seed regions and network information are provided in Table 4. We employed an LC metamask created by Dahl et al., which amalgamates six previously developed masks, averaging individual binarized masks at each voxel [44]. For the seeds used in the SN functional connectivity (FC) calculations, we used the seed regions of interest (ROI) provided by the CONN toolbox, specifically the right insular cortex within the SN [45]. During the first-level analysis, we computed seed-to-voxel connectivity maps for each participant, which were subsequently used in the group-level analysis. We used between-subjects contrasts [Male (1) Female (−1)] to evaluate the presence of any statistically significant differences in the LC and SN FC between the two sexes. Voxel-wise statistics were applied for the contrasts throughout the entire brain, with a threshold set at *p* < 0.05. In addition, corrections for the false discovery rate (FDR) at the cluster level and *p* < 0.001 at the uncorrected voxel level were implemented.

#### 4.4.3. Statistical Analyses

The R software (version 4.1.0) was utilized for the statistical analyses [46]. Demographic differences between males and females were evaluated using an independent two-sample *t*-test. Categorical variables, such as the carrier status of apolipoprotein ε4, were analyzed using the chi-square test. Furthermore, a general linear model was constructed with FC as the outcome variable and sex, global, and regional SUVR as independent variables. Additionally, the interaction effects of sex by regional and global SUVR on the LC and SN FC were estimated. All the statistical evaluations employed a two-tailed α level of 0.05 to define statistical significance.

## 5. Conclusions

This is one of the few studies that elucidated the sexual disparities of the LC and SN FC in preclinical AD. Differential patterns of LC FC and SN FC were observed in males and females with preclinical AD, which interacted with regional Aβ deposition. We believe sexual dimorphism should be considered in the LC-NE system and SN research on preclinical AD patients. Future studies explaining the role of LC FC in AD trajectory, with a more robust sample size, cognitive, and behavioral measurements, will be conducive to understanding the significance of the LC in AD research.

## Figures and Tables

**Table 1:** Demographic and clinical characteristics of the study participants (N = 89).

	Male (N = 37)	Female (N = 52)	*p* Value
**Age (years ± SD)**	69.3 ± 8.8	71.5 ± 7.9	0.218
**Education (years ± SD)**	13.4 ± 5.0	12.4 ± 2.7	0.287
**APOE4 carrier (Yes, %)**	32.4%	38.5%	0.719
**CERAD-K Battery (SD)**			
VF	15.7 ± 4.3	15.2 ± 4.5	0.605
BNT	12.8 ± 1.9	12.2 ± 1.9	0.174
MMSE	27.7 ± 2.0	27.5 ± 2.1	0.712
WLM	17.3 ± 3.2	18.6 ± 2.7	0.054
CP	10.5 ± 1.1	10.4 ± 0.9	0.767
WLR	5.8 ± 1.6	6.2 ± 1.6	0.278
WLRc	9.1 ± 1.1	9.4 ± 0.9	0.248
CR	7.4 ± 2.9	6.8 ± 2.7	0.329
**Global SUVR_PONS_**	0.7 ± 0.1	0.7 ± 0.1	0.788
**Regional SUVR_PONS_**			
ACC	0.7 ± 0.1	0.7 ± 0.1	0.624
FL	0.6 ± 0.1	0.6 ± 0.1	0.540
PL	0.5 ± 0.1	0.5 ± 0.1	0.937
PCC/Precuneus	0.7 ± 0.1	0.7 ± 0.1	0.702
TL	0.6 ± 0.1	0.6 ± 0.1	0.736

SD, standard deviation, MCI, mild cognitive impairment; APOE, Apolipoprotein E; CERAD-K, the Korean version of Consortium to Establish a Registry for Alzheimer’s Disease; VF, verbal fluency; BNT, 15-item Boston Naming Test; MMSE, Mini Mental Status Examination; WLM, word list memory; CP, constructional praxis; WLR, word list recall; WLRc, word list recognition; CR, constructional recall; SUVR_PONS_, standardized uptake value ratios of [18F] flutemetamol, with pons as a reference region; ACC, anterior cingulate cortex; FL, frontal lobes; PL, parietal lobes; PCC, posterior cingulate cortex; and TL, lateral temporal lobes.

**Table 2:** Regions showing interactive effect of sex and regional SUVR on locus coeruleus and salience network (voxel threshold: *p* < 0.001, uncorrected, cluster threshold: *p* < 0.05, cluster-size p-FDR corrected).

Seed Region and Network	Clusters (x,y,z)	Regions Covered by the Cluster	Cluster Size	Size p-FDR	Peak p-Uncorrected
Sex by frontal lobe SUVR interaction					
**Locus coeruleus**	+50, −28, +52	(1) Postcentral gyrus, right (2) Supramarginal gyrus, anterior division, right (3) Supramarginal gyrus, posterior division, right	50	0.000870	0.000007
**Salience network**	+20, +62, +02	(1) Frontal pole, right	35	0.025115	0.000013
	−18, −88, +40	(1) Lateral occipital cortex, superior division, left (2) Occipital pole left	30	0.028579	0.000033
**Sex by parietal lobe SUVR interaction**					
**Salience network**	+44, −70, +38	(1) Lateral occipital cortex, superior division, right	33	0.039429	0.000019
**Sex by temporal lobe SUVR interaction**					
**Salience network**	−08, −90, +34	(1) Lateral occipital cortex, superior division, left (2) Occipital pole, left (3) Cuneal cortex, left	32	0.038671	0.000073

FDR, false discovery rate; SUVR, standardized uptake value ratio.

**Table 3:** Regions showing interactive effect of sex and global SUVR on locus coeruleus and salience network (voxel threshold: *p* < 0.001, uncorrected, cluster threshold: *p* < 0.05, cluster-size p-FDR corrected).

Seed Region and Network	Clusters (x,y,z)	Regions Covered by the Cluster	Cluster Size	Size p-FDR	Peak p-Uncorrected
Sex by global SUVR interaction					
**Salience network**	−08, −88, +38	(1) Lateral occipital cortex, superior division, left (2) Occipital pole, left (3) Cuneal cortex, left	29	0.045532	0.000095
	−66, −26, +12	(1) Superior temporal gyrus, posterior division, left (2) Planum temporale, left	28	0.045532	0.000031

FDR, false discovery rate; SUVR, standardized uptake value ratio.

**Table 4:** Seed regions used for the resting-state functional connectivity analyses.

Seed Region and Network	Definition
**Locus coeruleus**	Amalgamation of six previously developed masks, averaging individual binarized masks at each voxel
**Salience network (insular cortex, right)**	MNI Coordinates (37,3,0)

## Data Availability

The data presented in this study are available on request from the corresponding author. The data are not publicly available due to institutional policies regarding data transfer.

## References

[1] Ibrahim B., Suppiah S., Ibrahim N., Mohamad M., Hassan H.A., Nasser N.S., Saripan M.I. (2021). Diagnostic power of resting-state fMRI for detection of network connectivity in Alzheimer’s disease and mild cognitive impairment: A systematic review. Hum. Brain Mapp..

[2] Ferretti M.T., Iulita M.F., Cavedo E., Chiesa P.A., Schumacher Dimech A., Santuccione Chadha A., Baracchi F., Girouard H., Misoch S., Giacobini E. (2018). Sex differences in Alzheimer disease—The gateway to precision medicine. Nat. Rev. Neurol..

[3] Pike C.J. (2017). Sex and the development of Alzheimer’s disease. J. Neurosci. Res..

[4] Radaghdam S., Karamad V., Nourazarian A., Shademan B., Khaki-khatibi F., Nikanfar M. (2021). Molecular mechanisms of sex hormones in the development and progression of Alzheimer’s disease. Neurosci. Lett..

[5] Guo L., Zhong M.B., Zhang L., Zhang B., Cai D. (2022). Sex Differences in Alzheimer’s Disease: Insights from the Multiomics Landscape. Biol. Psychiatry.

[6] Jack C.R., Bennett D.A., Blennow K., Carrillo M.C., Dunn B., Haeberlein S.B., Holtzman D.M., Jagust W., Jessen F., Karlawish J. (2018). NIA-AA Research Framework: Toward a biological definition of Alzheimer’s disease. Alzheimer’s Dement..

[7] Lin C., Ly M., Karim H.T., Wei W., Snitz B.E., Klunk W.E., Aizenstein H.J. (2020). The effect of amyloid deposition on longitudinal resting-state functional connectivity in cognitively normal older adults. Alzheimer’s Res. Ther..

[8] Sheline Y.I., Raichle M.E. (2013). Resting State Functional Connectivity in Preclinical Alzheimer’s Disease. Biol. Psychiatry.

[9] Schultz A.P., Chhatwal J.P., Hedden T., Mormino E.C., Hanseeuw B.J., Sepulcre J., Huijbers W., LaPoint M., Buckley R.F., Johnson K.A. (2017). Phases of Hyperconnectivity and Hypoconnectivity in the Default Mode and Salience Networks Track with Amyloid and Tau in Clinically Normal Individuals. J. Neurosci..

[10] Cavedo E., Chiesa P.A., Houot M., Ferretti M.T., Grothe M.J., Teipel S.J., Lista S., Habert M.-O., Potier M.-C., Dubois B. (2018). Sex differences in functional and molecular neuroimaging biomarkers of Alzheimer’s disease in cognitively normal older adults with subjective memory complaints. Alzheimer’s Dement..

[11] Heise V., Filippini N., Trachtenberg A.J., Suri S., Ebmeier K.P., Mackay C.E. (2014). Apolipoprotein E genotype, gender and age modulate connectivity of the hippocampus in healthy adults. NeuroImage.

[12] Chen Y., Chen T., Hou R. (2022). Locus coeruleus in the pathogenesis of Alzheimer’s disease: A systematic review. Alzheimer’s Dement. Transl. Res. Clin. Interv..

[13] Jacobs H.I.L., Becker J.A., Kwong K., Engels-Domínguez N., Prokopiou P.C., Papp K.V., Properzi M., Hampton O.L., d’Oleire Uquillas F., Sanchez J.S. (2021). In vivo and neuropathology data support locus coeruleus integrity as indicator of Alzheimer’s disease pathology and cognitive decline. Sci. Transl. Med..

[14] Prokopiou P.C., Engels-Domínguez N., Papp K.V., Scott M.R., Schultz A.P., Schneider C., Farrell M.E., Buckley R.F., Quiroz Y.T., El Fakhri G. (2022). Lower novelty-related locus coeruleus function is associated with Aβ-related cognitive decline in clinically healthy individuals. Nat. Commun..

[15] Luckey A.M., Robertson I.H., Lawlor B., Mohan A., Vanneste S. (2021). Sex Differences in Locus Coeruleus: A Heuristic Approach That May Explain the Increased Risk of Alzheimer’s Disease in Females. J. Alzheimer’s Dis..

[16] Zhang S., Hu S., Chao H.H., Li C.-S.R. (2016). Resting-State Functional Connectivity of the Locus Coeruleus in Humans: In Comparison with the Ventral Tegmental Area/Substantia Nigra Pars Compacta and the Effects of Age. Cereb. Cortex.

[17] Clewett D.V., Lee T.-H., Greening S., Ponzio A., Margalit E., Mather M. (2016). Neuromelanin marks the spot: Identifying a locus coeruleus biomarker of cognitive reserve in healthy aging. Neurobiol. Aging.

[18] Ross J.A., Alexis R., Reyes B.A.S., Risbrough V., Van Bockstaele E.J. (2019). Localization of amyloid beta peptides to locus coeruleus and medial prefrontal cortex in corticotropin releasing factor overexpressing male and female mice. Brain Struct. Funct..

[19] Chen H., Li W., Sheng X., Ye Q., Zhao H., Xu Y., Bai F., Alzheimer’s Disease Neuroimaging Initiative (2022). Machine learning based on the multimodal connectome can predict the preclinical stage of Alzheimer’s disease: A preliminary study. Eur. Radiol..

[20] Oschmann M., Gawryluk J.R. (2020). A Longitudinal Study of Changes in Resting-State Functional Magnetic Resonance Imaging Functional Connectivity Networks during Healthy Aging. Brain Connect..

[21] Zhang M., Guan Z., Zhang Y., Sun W., Li W., Hu J., Li B., Ye G., Meng H., Huang X. (2022). Disrupted coupling between salience network segregation and glucose metabolism is associated with cognitive decline in Alzheimer’s disease—A simultaneous resting-state FDG-PET/fMRI study. NeuroImage Clin..

[22] Leming M., Suckling J. (2021). Deep learning for sex classification in resting-state and task functional brain networks from the UK Biobank. NeuroImage.

[23] Rodberg E.M., den Hartog C.R., Dauster E.S., Vazey E.M. (2023). Sex-dependent noradrenergic modulation of premotor cortex during decision making. eLife.

[24] Ycaza Herrera A., Wang J., Mather M. (2019). The gist and details of sex differences in cognition and the brain: How parallels in sex differences across domains are shaped by the locus coeruleus and catecholamine systems. Prog. Neurobiol..

[25] Berridge C.W., Spencer R.C. (2016). Differential cognitive actions of norepinephrine a2 and a1 receptor signaling in the prefrontal cortex. Brain Res..

[26] Li C., Li Y., Zheng L., Zhu X., Shao B., Fan G., Liu T., Wang J., Alzheimer’s Disease Neuroimaging Initiative (2019). Abnormal Brain Network Connectivity in a Triple-Network Model of Alzheimer’s Disease. J. Alzheimer’s Dis..

[27] Brandes Lourenço R., Machado de Campos B., Rizzi L., Sakzenian de Souza M., Forlenza O.V., Giroud Joaquim H., Leme Talib L., Cendes F., Figueredo Balthazar M.L. (2022). Functional Connectome Analysis in Mild Cognitive Impairment: Comparing Alzheimer’s Disease Continuum and Suspected Non-Alzheimer Pathology. Brain Connect..

[28] He X., Qin W., Liu Y., Zhang X., Duan Y., Song J., Li K., Jiang T., Yu C. (2014). Abnormal salience network in normal aging and in amnestic mild cognitive impairment and Alzheimer’s disease. Hum. Brain Mapp..

[29] Wang S., Sun H., Hu G., Xue C., Qi W., Rao J., Zhang F., Zhang X., Chen J. (2021). Altered Insular Subregional Connectivity Associated With Cognitions for Distinguishing the Spectrum of Pre-clinical Alzheimer’s Disease. Front. Aging Neurosci..

[30] Hojjati S.H., Ebrahimzadeh A., Babajani-Feremi A. (2019). Identification of the Early Stage of Alzheimer’s Disease Using Structural MRI and Resting-State fMRI. Front. Neurol..

[31] Ueno D., Matsuoka T., Kato Y., Ayani N., Maeda S., Takeda M., Narumoto J. (2020). Individual Differences in Interoceptive Accuracy Are Correlated With Salience Network Connectivity in Older Adults. Front. Aging Neurosci..

[32] Prentice F., Murphy J. (2022). Sex differences in interoceptive accuracy: A meta-analysis. Neurosci. Biobehav. Rev..

[33] Papanikolaou A., Rodrigues F.R., Holeniewska J., Phillips K.G., Saleem A.B., Solomon S.G. (2022). Plasticity in visual cortex is disrupted in a mouse model of tauopathy. Commun. Biol..

[34] Song Y., Wu H., Chen S., Ge H., Yan Z., Xue C., Qi W., Yuan Q., Liang X., Lin X. (2022). Differential Abnormality in Functional Connectivity Density in Preclinical and Early-Stage Alzheimer’s Disease. Front. Aging Neurosci..

[35] Jeong J.-K., Tremere L.A., Burrows K., Majewska A.K., Pinaud R. (2011). The Mouse Primary Visual Cortex Is a Site of Production and Sensitivity to Estrogens. PLoS ONE.

[36] Moon Y., Lim C., Kim Y., Moon W.-J. (2021). Sex-Related Differences in Regional Blood–Brain Barrier Integrity in Non-Demented Elderly Subjects. Int. J. Mol. Sci..

[37] Mahoney J.R., Blumen H.M., De Sanctis P., Fleysher R., Frankini C., Hoang A., Hoptman M.J., Jin R., Lipton M., Nunez V. (2023). Visual-somatosensory integration (VSI) as a novel marker of Alzheimer’s disease: A comprehensive overview of the VSI study. Front. Aging Neurosci..

[38] Lyu J., Kim H.-Y. (2016). Gender-Specific Incidence and Predictors of Cognitive Impairment among Older Koreans: Findings from a 6-Year Prospective Cohort Study. Psychiatry Investig..

[39] Lee J.H., Lee K.U., Lee D.Y., Kim K.W., Jhoo J.H., Kim J.H., Lee K.H., Kim S.Y., Han S.H., Woo J.I. (2002). Development of the Korean version of the Consortium to Establish a Registry for Alzheimer’s Disease Assessment Packet (CERAD-K): Clinical and neuropsychological assessment batteries. J. Gerontol. B Psychol. Sci. Soc. Sci..

[40] Park J.H. (1989). Standardization of Korean of the mini-mental state examination (MMSE-K) for use in the elderly. Part II. Diagnostic validity. J. Korean Neuropsychiatr. Assoc..

[41] Morris J.C. (1997). Clinical dementia rating: A reliable and valid diagnostic and staging measure for dementia of the Alzheimer type. Int. Psychogeriatr..

[42] Thurfjell L., Lilja J., Lundqvist R., Buckley C., Smith A., Vandenberghe R., Sherwin P. (2014). Automated quantification of 18F-flutemetamol PET activity for categorizing scans as negative or positive for brain amyloid: Concordance with visual image reads. J. Nucl. Med..

[43] Whitfield-Gabrieli S., Nieto-Castanon A. (2012). Conn: A functional connectivity toolbox for correlated and anticorrelated brain networks. Brain Connect..

[44] Dahl M.J., Mather M., Werkle-Bergner M., Kennedy B.L., Guzman S., Hurth K., Miller C.A., Qiao Y., Shi Y., Chui H.C. (2022). Locus coeruleus integrity is related to tau burden and memory loss in autosomal-dominant Alzheimer’s disease. Neurobiol. Aging.

[45] Whitfield-Gabrieli S., Moran J.M., Nieto-Castañón A., Triantafyllou C., Saxe R., Gabrieli J.D.E. (2011). Associations and dissociations between default and self-reference networks in the human brain. NeuroImage.

[46] R Core Team R: A Language and Environment for Statistical Computing. https://www.r-project.org.

